# Achievability of 3D planned bimaxillary osteotomies: maxilla-first versus mandible-first surgery

**DOI:** 10.1038/s41598-017-09488-4

**Published:** 2017-08-24

**Authors:** Jeroen Liebregts, Frank Baan, Martien de Koning, Edwin Ongkosuwito, Stefaan Bergé, Thomas Maal, Tong Xi

**Affiliations:** 1 Department of Oral and Maxillofacial Surgery, Radboud University Nijmegen Medical Centre, Geert Grooteplein 10, 6525 GA Nijmegen, The Netherlands; 2 Department of Orthodontics and Craniofacial Biology, Radboud University Nijmegen Medical Centre, Philips van Leydenlaan 25, 6525 EX Nijmegen, The Netherlands

## Abstract

The present study was aimed to investigate the effects of sequencing a two-component surgical procedure for correcting malpositioned jaws (bimaxillary osteotomies); specifically, surgical repositioning of the upper jaw—maxilla, and the lower jaw—mandible. Within a population of 116 patients requiring bimaxillary osteotomies, the investigators analyzed whether there were statistically significant differences in postoperative outcome as measured by concordance with a preoperative digital 3D virtual treatment plan. In one group of subjects (n = 58), the maxillary surgical procedure preceded the mandibular surgery. In the second group (n = 58), the mandibular procedure preceded the maxillary surgical procedure. A semi-automated analysis tool (OrthoGnathicAnalyser) was applied to assess the concordance of the postoperative maxillary and mandibular position with the cone beam CT-based 3D virtual treatment planning in an effort to minimize observer variability. The results demonstrated that in most instances, the maxilla-first surgical approach yielded closer concordance with the 3D virtual treatment plan than a mandibular-first procedure. In selected circumstances, such as a planned counterclockwise rotation of both jaws, the mandible-first sequence resulted in more predictable displacements of the jaws.

## Introduction

Over the last decades, the surgical approach used during bimaxillary surgery, either maxillary-first or mandibular-first sequence, has been a controversial topic in the field of orthognathic surgery (corrective jaw surgery). The traditional approach is to operate on the maxilla first. Notably, several recent publications have described encouraging results with the mandible first sequencing protocol^1–3^, with Perez and Ellis demonstrating the benefit of this approach in patients with counterclockwise (CCW) mandibular rotation and downgrafting of the posterior maxilla^4^. Despite these recent reports, there is still limited clinical and scientific consensus on which surgical approach taken during corrective jaw surgery can provide the most predictable clinical result.

Recently, computer-assisted 3D virtual surgical planning has been shown to increase the predictability of the postoperative outcomes, and has increasingly become the standard approach for complex orthognathic reconstructions^5^. The standard approach to transfer the virtual planning from the computer to the patient during surgery, is the use of 3D printed interocclusal splints. These splints act as an important control in order to correctly position the maxilla and mandible in the planned positions^5–7^.

A key consideration when using 3D virtual surgical planning is the accuracy of the system in obtaining the planned repositioning of the maxilla and mandible. Therefore, several analytic tools, such as the OrthoGnathicAnalyser (OGA), have been developed to evaluate the surgical accuracy of the computer-assisted surgical approach robustly. Compared to conventional cephalometry, OGA is able to quantify the concordance of the postoperative maxillary and mandibular positions with the 3D surgical planning without the need to identify anatomical landmarks multiple times, thereby minimizing the observer variability. With OGA, the accuracy of 3D planning and surgical outcome of bimaxillary surgery with different sequencing protocols can be analysed in an objective, reproducible and systematic way^8^.

The aim of this study was to investigate the effects of sequencing bimaxillary osteotomies, maxilla-first versus mandible-first surgical protocol, on the achievability of the 3D virtually planned repositioning of the maxilla and mandible.

## Results

In this cohort study, 116 consecutive patients were enrolled (80 female (69%); 36 male (31%)), with a mean age at surgery of 28 years (range 16–57 years; Table 2). Of the 116 patients who underwent bimaxillary surgery, 58 patients (50%) were operated using the maxilla-first protocol whereas the other 58 patients (50%) were treated with the mandible-first surgical protocol. Before bimaxillary surgery, 33 patients underwent a surgically assisted rapid maxillary expansion (15 in the maxilla-first group and 18 in the mandible-first group), with an additional 61 patients undergoing a genioplasty during surgery (33 in the maxilla-first group and 28 in the mandible-first group). As shown in Table 2, no differences within patient related factors and surgical displacements were found between the maxilla-first and mandible-first groups.

**Table 2:** Patient demographics Age, gender and surgical difference distribution within the study population.

		Maxillary first Surgery	Mandible first Surgery
Population (n = 116)		58	58
Age	Mean	28.6	27.5
	SD	11.0	10.6
	Range	16–57	16–55
Male (n = 36)		15	21
Female (n = 80)		43	37
SARME in history		15	18
Genioplasty		33	28

SD: Standard deviation.

### Planned values

No significant differences in surgical displacement (translations and rotations) of the bimaxillary complex were found between the maxilla-first and mandible-first groups, except for the direction and magnitude of maxillary pitch (Table 3). In the maxilla-first group a mean pitch of 1.98° in the clockwise (CW) direction was found whereas the mean pitch in the mandible-first group was 0.39° in the CCW direction (p = 0.01).

**Table 3:** Planned surgical translations and rotations of the maxilla between maxilla-first and mandible-first patients.

		Maxillary first Mean ± SD	Mandible first Mean ± SD	P-value
Maxillary Translations (mm)	Anterior/Posterior	3.90 ± 1.73	4.35 ± 1.83	0.17
	Left/Right	−0.04 ± 1.68	−0.16 ± 1.42	0.67
	Cranial/Caudal	−0.45 ± 2.91	−0.13 ± 2.91	0.56
Maxillary Rotations(°)	Pitch	1.98 ± 3.91	−0.39 ± 0.5.99	0.01
	Roll	−0.30 ± 2.05	−0.07 ± 1.99	0.53
	Yaw	0.07 ± 1.48	0.14 ± 1.42	0.78
Mandibular Translations (mm)	Anterior/Posterior	−8.02 ± 5.69	−7.32 ± 4.58	0.47
	Left/Right	−0.05 ± 2.54	−0.35 ± 2.44	0.21
	Cranial/Caudal	1.07 ± 3.12	0.36 ± 3.68	0.26
Mandibular Rotations(°)	Pitch	−0.11 ± 4.57	−1.21 ± 6.46	0.29
	Roll	−0.32 ± 2.24	−0.08 ± 2.30	0.57
	Yaw	0.07 ± 2.57	−0.07 ± 2.64	0.57

Translation Anterior/Posterior: a positive value means that the maxilla was planned anteriorly, a negative value means that the maxilla was planned posteriorly. Translation Left/Right: a positive value means that the maxilla was planned to the right, a negative value means that the maxilla was planned to the left. Translation Cranial/Caudal: a positive value means that the maxilla was planned cranially, a negative value means that the maxilla was planned caudally. Rotation Pitch: a positive value means a counterclockwise rotation, a negative value means a clockwise rotation. Rotation Roll: a positive value means a counterclockwise rotation around the horizontal axis, a negative value means a clockwise rotation around the horizontal axis. Rotation Yaw: a positive value means a counterclockwise rotation around the vertical axis, a negative value means a clockwise rotation around the vertical axis. Data presented as means ± SD.

### Overall achievability

**Figure 1:**
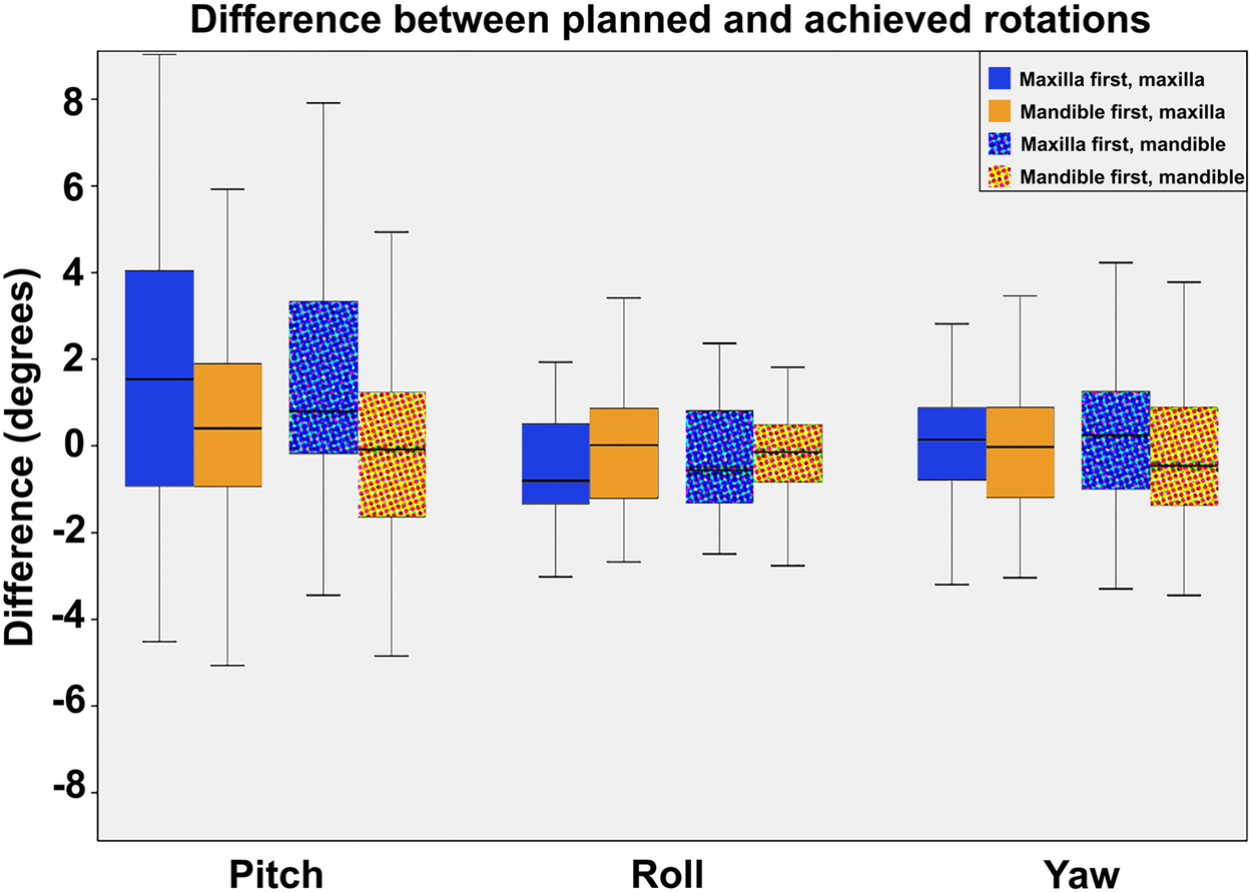
Boxplot of the differences between planned rotations and the postoperative outcome for the mandible and maxilla. Both the maxilla- and mandible-first groups are displayed in the boxplot. The whiskers of the boxplot represent the 25^th^ and 75^th^ percentiles. For the pitch the largest deviation is seen, the smallest deviation is seen in the roll. A negative pitch means that the achieved pitch is larger than the planned pitch, the same applies for the roll and yaw.

**Figure 2:**
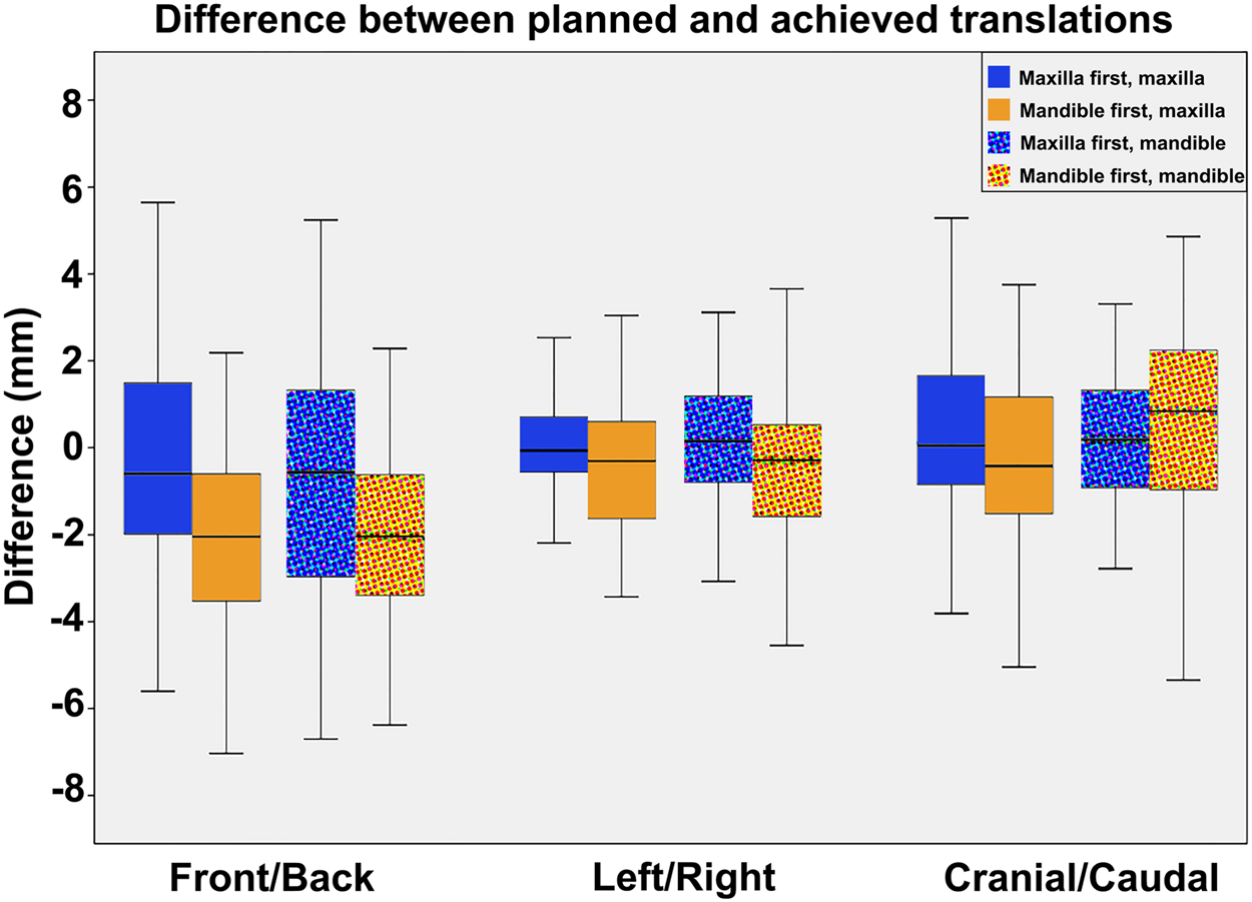
Boxplot of the differences between planned translations and the postoperative outcome for the mandible and maxilla. Both the maxilla- and mandible-first groups are displayed in the boxplot. The whiskers of the boxplot represent the 25^th^ and 75^th^ percentiles. For the front/back translation the largest deviation is seen, the smallest deviation is seen in the left/right translation. A negative front/back translation means that the achieved front/back translation is larger than the planned front/back translation, the same applies for the left/right and cranial/caudal translation.

The overall comparison between the pre-surgical 3D planning to the measured rotation and translations of the bimaxillary complex after surgery, in the maxilla-first and mandible-first groups, are displayed in Tables 4 and 5, respectively. In both groups, the achieved pitch showed the largest deviation compared to the 3D planning, whereas the achieved roll showed the least deviation (Fig. 1). Concerning the translational displacement, the anterior/posterior displacement was the least accurate whereas the left/right displacement deviated the least from the 3D planning in both the maxilla-first and mandible-first group (Fig. 2).

**Table 4:** Surgical planned, realized and difference values for the maxilla in both mandible- and maxilla first patients. Translations are given in millimeters, rotations are given in degrees.

		Maxilla first					Mandible first					
		n	Planned Mean ± SD	Realized Mean ± SD	Difference Mean ± SD	p-value*	n	Planned Mean ± SD	Realized Mean ± SD	Difference Mean ± SD	p-value*	p-value**
**Translation**												
X	Left	18	1.91 ± 0.86	1.84 ± 1.46	0.07 ± 1.02	0.79	33	1.25 ± 1.09	0.37 ± 1.37	0.88 ± 1.52	0.01	0.06
	Right	14	2.28 ± 0.84	1.66 ± 1.81	0.62 ± 1.62	0.19	20	0.00 ± 0.00	0.81 ± 1.68	0.81 ± 1.68	0.27	0.58
	None	26	0.00 ± 0.00	0.07 ± 1.19	−0.07 ± 1.19	0.78	5	1.10 ± 0.85	0.80 ± 1.19	0.30 ± 1.40	0.14	0.05
Y	Anterior	56	4.04 ± 1.58	3.58 ± 2.51	0.45 ± 2.52	0.28	58	4.35 ± 1.81	2.39 ± 2.04	1.97 ± 1.86	0.00	0.00
	Posterior	0	—	—	—	—	0	—	—	—	—	—
	None	2	0.00 ± 0.00	5.17 ± 0.47	−5.17 ± 0.47	—	0	—	—	—	—	—
Z	Cranial	23	2.78 ± 1.67	3.16 ± 2.47	0.38 ± 2.04	0.39	31	2.06 ± 1.66	1.89 ± 2.32	0.17 ± 2.20	0.67	0.36
	Caudal	33	2.47 ± 1.33	2.31 ± 2.59	0.16 ± 1.98	0.65	26	2.74 ± 1.70	2.90 ± 1.96	−0.16 ± 1.23	0.52	0.48
	None	2	0.00 ± 0.00	5.13 ± 1.07	5.13 ± 1.07	0.13	1	0.00 ± 0.00	−0.94 ± 0.00	−0.94 ± 0.00	—	—
**Rotation**												
Pitch	CW	32	4.78 ± 2.04	2.82 ± 3.26	1.96 ± 2.73	0.00	27	5.35 ± 2.89	3.04 ± 2.46	2.48 ± 1.90	0.00	0.59
	CCW	9	4.21 ± 2.90	3.10 ± 3.01	1.11 ± 1.60	0.04	29	5.76 ± 3.38	3.99 ± 2.78	1.77 ± 2.63	0.00	0.62
	None	17	0.00 ± 0.00	1.92 ± 2.81	−1.92 ± 2.81	0.02	2	0.00 ± 0.00	−0.16 ± 0.79	−0.16 ± 0.79	0.57	0.09
Roll	CW	22	2.10 ± 1.36	1.26 ± 1.55	0.84 ± 1.39	0.02	29	1.94 ± 1.41	1.18 ± 1.02	0.76 ± 1.23	0.01	0.98
	CCW	11	2.60 ± 1.76	2.95 ± 2.42	0.35 ± 1.22	0.21	24	1.93 ± 1.36	1.41 ± 1.31	0.52 ± 1.62	0.26	0.11
	None	25	0.00 ± 0.00	0.33 ± 0.98	−0.33 ± 0.98	0.11	5	0.00 ± 0.00	0.04 ± 0.97	0.04 ± 0.97	0.92	0.26
Yaw	CW	17	1.36 ± 1.07	1.00 ± 1.58	0.36 ± 1.69	0.32	28	1.30 ± 0.90	0.41 ± 2.00	0.89 ± 1.67	0.05	0.38
	CCW	14	1.93 ± 1.23	0.96 ± 1.70	0.97 ± 1.51	0.16	27	1.17 ± 1.22	0.53 ± 1.39	0.64 ± 1.47	0.04	0.94
	None	27	0.00 ± 0.00	0.04 ± 1.14	−0.04 ± 1.14	0.50	3	0.00 ± 0.00	0.39 ± 1.21	−0.39 ± 1.21	0.25	0.17

Translation Anterior/Posterior: a positive value means that the mandible was positioned more posteriorly than planned, a negative value means that the mandible was positioned more anteriorly than planned. Translation Left/Right: a positive value means that the mandible was positioned more to the right compared to the planning, a negative value means that the mandible was positioned more to the left compared to the planning. Translation Cranial/Caudal: a positive value means that the mandible was displaced more cranially compared to the planning, a negative value means that the mandible was displaced more cranially compared to the planning. Rotation Pitch: a positive value means an anti-clockwise rotation compared to the planning, a negative value means a clockwise rotation compared to the planning. Rotation Roll: a positive value means an anti-clockwise rotation around the horizontal axis compared to the planning, a negative value means a clockwise rotation around the horizontal axis compared to the planning. Rotation Yaw: a positive value means an anti-clockwise rotation around the vertical axis compared to the planning, a negative value means a clockwise rotation around the vertical axis compared to the planning. Data presented as means ± SD.

CW: Clockwise, CCW: Counterclockwise. *p-value of the difference between the planned and realized values, **p-value of the difference of the discrepancies (planned-realized values) between the maxilla-first group and mandible-first group.

### Predictability of translational displacements

In the maxilla-first group, the achievability of the anterior displacement of the maxilla was significantly higher, compared to the mandible-first group (Table 4). A discrepancy between the planning and the postoperative result of 0.45 ± 2.52 mm versus 1.97 ± 1.86 mm respectively (p < 0.01) was found. The achieved anterior displacement was smaller than the planned movement in almost all cases. Concerning the anterior positioning of the mandible, a similar association was found, in favour of the maxilla-first group (0.73 ± 2.65 mm versus 2.11 ± 2.13 mm respectively, p = 0.01). The precision of the mandibular positioning was lower than the maxilla in both groups. Neither statistical significant differences nor clinical relevant differences in the achievability of the vertical and transverse displacements were found between the maxilla-first and mandible-first groups.

### Predictability of rotational displacements

With regard to rotational displacements, the CW pitch of the mandible was attained more precisely in the mandible-first group compared to the maxilla-first group, a discrepancy of 1.30 ± 2.20° versus 3.10 ± 2.83° (p = 0.02) was found (Table 5). No significant difference between the achievability of a CW maxillary pitch and a CCW maxillary pitch was present between the two groups (p = 0.07). In the maxilla-first group, a CW pitch was more difficult to accomplish than a CCW pitch, resulting in an inaccuracy of 1.96° and 1.11° respectively. The mean discrepancies between the planned and achieved roll and yaw of the maxilla and mandible were below 1.2° in both groups.

**Table 5:** Surgical planned, realized and difference values for the mandible in both mandible and maxilla first patients.

		Maxilla first						Mandible first					
		n	Planned Mean ± SD		Realized Mean ± SD	Difference Mean ± SD	p-value*	n	Planned Mean ± SD	Realized Mean ± SD	Difference Mean ± SD	p-value*	p-value**
**Translation**													
X	Left	33	1.34 ± 0.98	1.03 ± 2.23		0.31 ± 1.97	0.39	31	2.06 ± 1.64	0.93 ± 1.94	1.13 ± 1.54	0.00	0.06
	Right	20	2.91 ± 2.64	2.00 ± 3.13		0.91 ± 1.82	0.02	27	1.61 ± 1.49	1.34 ± 1.46	0.27 ± 1.46	0.36	0.14
	None	5	0.00 ± 0.00	0.56 ± 1.11		−0.56 ± 0.11	0.84	0	—	—	—	—	—
Y	Anterior	50	9.61 ± 4.23	8.88 ± 4.96		0.73 ± 2.65	0.06	52	8.28 ± 3.73	6.17 ± 3.44	2.11 ± 2.13	0.00	0.01
	Posterior	6	2.56 ± 2.04	4.83 ± 3.25		2.27 ± 2.01	0.05	6	1.03 ± 0.44	2.50 ± 0.74	1.47 ± 1.05	0.03	0.45
	None	2	0.00 ± 0.00	5.79 ± 5.79		−5.79 ± 5.79	—	0	—	—	—	—	—
Z	Cranial	33	3.25 ± 2.03	2.56 ± 3.09		0.69 ± 2.16	0.08	34	2.94 ± 1.43	1.79 ± 2.74	1.16 ± 2.40	0.01	0.52
	Caudal	20	2.23 ± 1.37	2.44 ± 1.98		−0.21 ± 2.00	0.61	24	3.29 ± 2.53	3.35 ± 2.60	−0.05 ± 1.98	0.90	0.84
	None	5	0.00 ± 0.00	1.38 ± 3.19		1.38 ± 3.19	0.50	0	—	—	—	—	—
**Rotation**													
Pitch	CW	27	3.56 ± 3.11	0.46 ± 3.63		3.10 ± 2.83	0.00	21	5.75 ± 3.90	4.45 ± 3.74	1.30 ± 2.20	0.02	0.02
	CCW	30	3.66 ± 2.63	3.52 ± 3.46		0.15 ± 2.53	0.74	37	5.16 ± 3.53	4.21 ± 3.04	0.95 ± 2.08	0.01	0.10
	None	1	0.00 ± 0.00	6.75 ± 1.17		−6.75 ± 1.17	—	0	—	—	—	—	—
Roll	CW	33	1.66 ± 1.61	1.03 ± 1.67		0.71 ± 1.11	0.00	35	1.47 ± 1.38	0.90 ± 1.28	0.56 ± 0.97	0.02	0.86
	CCW	24	1.51 ± 1.63	1.44 ± 2.15		0.07 ± 1.24	0.80	23	2.03 ± 1.70	1.39 ± 1.50	0.64 ± 1.15	0.02	0.13
	None	1	0.00 ± 0.00	0.21 ± 1.13		−0.21 ± 1.13	—	0	—	—	—	—	—
Yaw	CW	29	1.62 ± 1.69	0.93 ± 2.09		0.72 ± 1.40	0.01	29	2.04 ± 1.77	0.85 ± 1.46	1.19 ± 1.56	0.00	0.21
	CCW	28	2.09 ± 1.75	0.77 ± 1.90		1.40 ± 1.94	0.00	29	1.90 ± 1.68	1.10 ± 1.24	0.81 ± 0.61	0.01	0.29
	None	1	0.00 ± 0.00	0.55 ± 0.55		0.55 ± 0.61	—	0	—	—	—	—	—

Translations are given in millimeters, rotations are given in degrees. Translation Anterior/Posterior: a positive value means that the mandible was positioned more posteriorly than planned, a negative value means that the mandible was positioned more anteriorly than planned. Translation Left/Right: a positive value means that the mandible was positioned more to the right compared to the planning, a negative value means that the mandible was positioned more to the left compared to the planning. Translation Cranial/Caudal: a positive value means that the mandible was displaced more cranially compared to the planning, a negative value means that the mandible was displaced more cranially compared to the planning. Rotation Pitch: a positive value means an anti-clockwise rotation compared to the planning, a negative value means a clockwise rotation compared to the planning. Rotation Roll: a positive value means an anti-clockwise rotation around the horizontal axis compared to the planning, a negative value means a clockwise rotation around the horizontal axis compared to the planning. **R**otation Yaw: a positive value means an anti-clockwise rotation around the vertical axis compared to the planning, a negative value means a clockwise rotation around the vertical axis compared to the planning. Data presented as means ± SD.

CW: Clockwise, CCW: Counterclockwise. *p-value of the difference between the planned and realized values, **p-value of the difference of the discrepancies (planned-realized values) between the maxilla-first group and mandible-first group.

## Discussion

The sequence of osteotomies in bimaxillary surgery has been debated frequently in the field of orthognathic surgery^9^. To date, a limited number of studies have been conducted on the accuracy of sequence in bimaxillary osteotomies^1–4^. This is first 3D clinical cohort study, to our knowledge, which evaluates the influences of maxilla-first or mandible-first surgical sequence of bimaxillary osteotomies on the achievability of the 3D virtual treatment planning.

The strength of the present study is not only the use of 3D CBCT data to carry out the preoperative 3D planning and postoperative evaluation, but also the application of the newly designed and validated tool, the OGA^8^. In contrast to all conventional 2D and 3D cephalometric analyses, the OGA eliminates the necessity of identifying anatomical landmarks multiple times. By overcoming the landmark identification error, the OGA is an observer independent, semi-automatic tool, which is able to analyse the accuracy of the 3D planning and surgical outcome in an objective, reproducible and systematic way. Thus, it is now possible to identify and quantify small 3D-translational and -rotational discrepancies in the jaw position between two CBCTs.

The ideal study design to evaluate the influence of sequencing bimaxillary osteotomies and the achievability of 3D planning is a randomized controlled trial, having patients who are randomly assigned to the maxilla-first and mandible-first groups, while controlling all possible covariates. However, in clinical practice, this ideal study design may encounter grave ethical issues. Therefore, a retrospective cohort study was set up. The clinical protocol and principles of 3D planning were identical in both groups. The sequence of bimaxillary surgery was only dependent on the year of operation and not influenced by covariates. In this way, the selection bias was kept to a minimum. This is reflected by the fact that all patient characteristics and surgical movements did not differ significantly between the maxilla-first and mandible-first groups (except for the pitch of maxilla).

Our results have demonstrated that the positioning of the bimaxillary complex is generally more accurate when the maxilla is operated first, especially in the anterior displacement of the jaws. These results differ from those presented by Ritto *et al*.^3^ who has stated that the both the maxilla-first and mandible-first surgery could provide a reliable outcome. As the mean displacement of the maxilla was comparable between the two studies (4 mm), we believe that the difference is caused by the use of cephalograms and conventional cephalometry. The measurement errors in our study are significantly reduced by applying the state-of-art 3D imaging technology, thereby revealing the true underlying differences between the maxilla-first and mandible-first groups.

The double seating of the condyles in the mandible-first group could have induced more inaccuracies. During surgery the condyles are prone to displacements in their fossa as the result of manipulations of the proximal segments during fixation of the distal mandibular segment^10^. Small discrepancies in the condylar seating (1 mm or less) can create significant occlusal interferences, leading to significant deviations in jaw positioning^3^. This heavily impacts the mandible-first surgical approach, as the condylar seating needs to be performed twice, which not only affects the mandibular positioning, but also the maxillary positioning. Therefore, the overall accuracy of the bimaxillary positioning is in favour of the maxilla-first sequence, in which the condylar seating is only carried out once (only influencing the mandibular positioning). Thus, a better accuracy of the maxillary positioning is achieved compared to mandibular positioning, in both the maxilla-first and mandible-first groups.

Previous studies^1, 3, 4, 11^ have described that mistakes in the transfer of the correct mandibular position by face-bow registration to the articulator, will lead to an incorrect maxillary position. Therefore, the mandibular-first surgery is more accurate as it is less prone to errors caused by the incorrect mounting of the inferior model. By using a 3D-CBCT-based operation planning, coupled with the use of a wax bite to seat the condyles in centric relationship during CBCT scanning, the condyle-fossa remains stable throughout the planning process^12^. This eliminates the need to correct for inaccuracies in the planning phase by using a mandible-first surgical approach, as the preoperative planning of bimaxillary osteotomies is optimized with the 3D planning. Thus, in combination with 3D virtual planning of orthognathic surgery, the inherent advantages of maxilla-first sequence prevail.

The pitch showed the largest rotational deviation of all displacements. This can be the cause of bone interferences between the pterygoid plates and the osteotimized posterior maxilla. Intraoperatively it is hard to visually check for bone interferences in the posterior maxilla. This can result in premature bone contacts and lead to a deviation in the pitch. Another reason for the larger inaccuracies in the pitch might be the non-centric relation of the mandible when the interocclusal splint is used to set the maxilla in to its desired position^1^.

In line with the findings of Hsu *et al*.^13^ a lower achievability of the preoperative planning is seen in both the maxilla-first and mandible-first groups for the translations and rotations of the mandible compared to the maxilla. As this finding is present in both groups, a possible cause can be the positioning of the mandible during postoperative CBCT scanning. The postoperative CBCT scan was acquired without the interposition of the final splint. This may lead to a different occlusion than planned due to occlusal interferences, causing a larger discrepancy between the 3D planned and actually achieved mandibular position. In addition, the neuromuscular is not yet accomplished one week following bimaxillary surgery. As the CBCT scans were acquired without the use of elastics, traction from soft tissues surrounding the bimaxillary complex would have caused displacement of the mandible in the opposite direction to the surgical movements during the scanning process. Therefore, the concordance between the postoperative mandibular position and the 3D planning was inferior to that of the maxillary position. The position of the maxilla was not affected by the presence of the splint, elastics nor the occlusion.

The results from the present study highlighted the correlation between the magnitude of the translational and rotational movements, and the achievability of the 3D planning. The planned translations and rotations of more than 4 mm or 4 degrees showed a significant larger discrepancy between planning and post-operative outcome compared to cases with a smaller translations and rotations, particularly in the left/right translations and pitch of the maxilla (p < 0.03 and p < 0.01) and yaw of the mandible (p < 0.01). These findings are in line with the study of Semaan and Goonewardene^14^. In their analyses of the accuracy of Le Fort I surgeries, a greater maxillary advancement tended to be accompanied by more inaccuracies, such as an over-rotation of the maxilla in cases of a CW pitch and a maxillary under-rotation if a CCW pitch was planned. Soft tissue traction on the maxilla and the positioning of the osteotomy line would have influenced the accuracy of surgery.

While the maxilla first sequence is generally preferred^1, 15^ and found to be more accurate in this study, the mandible first sequence would be favoured in two specific situations:

1. CCW rotation of the bimaxillary complex
  In contrast to the anterior maxillary and mandibular displacements, the mandible-first operating sequence was able to achieve a CCW pitch of the mandible which was more accurate compared to the maxilla-first protocol. This objective finding has confirmed what Perez and Ellis^4^ postulated earlier, that it is preferable to operate the mandible first when performing a CCW rotation of the bimaxillary complex. In cases of maxilla-first sequence, when a CCW of the maxilla is required, the interocclusal wafer is thicker anteriorly than posteriorly, making the intermaxillary fixation more difficult to manage. In addition, there is little bony support of the posterior maxilla which makes the positioning and fixation of the maxilla more prone to errors. Since the fixation of a CCW pitched maxilla is more challenging and since the maxilla is subdued to reactive forces during the subsequent mandibular osteotomy and fixation, the stability and predictability would be greater when the mandible would be operated first^15^.
2. Segmental Le Fort I osteotomies

Cottrell and Wolford described that the mandible-first sequence would make complex dual-arch bimaxillary osteotomies, such as segmental Le Fort I osteotomies, more reliable by avoiding tension on the repositioned maxilla during the mandibular surgery 15. When performing the segmental Le Fort I osteotomies prior to mandibular surgery, traction on the weakened maxillary segments is exerted, which may influence the position of the segments and the subsequent mandibular position. By inverting the sequence, the maxillary segments can be positioned using the splint that is firmly attached to the newly positioned mandible, facilitating a more stable positioning of the maxillary segments. As all maxillas were operated in one-piece in the present study, this possible advantage of a mandible-first sequence could not be observed. Future 3D clinical studies are recommended to provide evidence for this sequencing protocol in segmental Le Fort I osteotomies. As long as a stable fixation of the maxilla can be achieved, the maxilla-first sequence should considered to be the first choice.

It should be underlined that when performing a mandible-first surgery, the mandibular osteotomies should be carried out meticulously and a stable fixation of the mandibular segments is required. In cases of a bad-split during mandibular surgery, an accurate positioning of the maxilla can be very challenging. The management of such unforeseen events in cases of mandible-first surgery should be in experienced hands.

An important step in the 3D virtual planning of bimaxillary osteotomies is the “virtual mandibular autorotation” which is in some cases required. An example is the downgrafting of the maxilla in the maxilla-first sequence. A realistic autorotation of the mandible is required to predict the subsequent mandibular position and the required movements of the mandible in order to create a harmonious facial profile. When a large CW or CCW rotation of the mandible is planned in the mandible-first sequence, the virtual mandible will also undergo “virtual mandibular autorotation”. Up till today, the virtual autorotation is still a weak point in the 3D virtual surgery planning as “virtual mandibular autorotation” is based on one single rotation over a predefined axis through both condyles. In reality, the autorotation of the mandible is a combination of rotational and translational movements of the condyles. Therefore, this should be one of the focuses of future studies on the accuracy of 3D virtual orthognathic planning.

Whether the maxilla or mandible should be operated first is still up to the surgeon to decide. The surgeons experience and preferences play an important role in the choice for maxilla-first or mandible-first surgery. While there appears to be advantages to support the use of mandibular-first sequence in specific cases, future prospective studies on its reliability, accuracy, and short- and long-term outcomes are required. As the present study has provided evidence for a superior predictability of maxilla-first surgery, the authors recommend the use of maxilla-first sequencing protocol unless a CCW pitch of the mandible is planned.

The sequence of bimaxillary osteotomies influences the achievability of the 3D virtual operation planning significantly. With maxilla-first surgery, the 3D planned translational and rotational movements of the maxilla and mandible can be accomplished more accurately compared to mandible-first surgery. However, in cases of bimaxillary CCW pitch, the mandible-first surgery is preferred. 3D virtual planning in combination with an optimised sequencing of osteotomies provide highly predictable results in bimaxillary surgery.

## Patients and Methods

Patients who underwent bimaxillary osteotomies consecutively in the period from 2010 to 2014 at the Department of Oral and Maxillofacial Surgery in Radboud University Nijmegen Medical Centre were included in this study. The inclusion criteria were a non-syndromatic dysgnathia requiring bimaxillary osteotomies with or without genioplasty and the availability of a CBCT scan before and directly after surgery. All patients received preoperative orthodontic treatment to align their teeth and had a minimum of 24 teeth. The exclusion criteria were the usage of a chin support during CBCT-scanning, previous history of facial trauma with fractures of facial bones, or a history of orthognatic surgery, with the exception of a SARME (Surgically Assisted Rapid Maxillary Expansion) procedure.

This study was conducted in accordance with the World Medical Association Declaration of Helsinki on medical research ethics. The approval of this study was granted by the Institutional Review Board (CMO Arnhem-Nijmegen, #181/2005) and informed consent were obtained for this study. All patient data were anonymized and de-identified prior to analysis.

### Data acquisition

CBCT scans were acquired four weeks prior to surgery and within one week after bimaxillary surgery using a standard CBCT scanning protocol (i-CAT, 3D Imaging System, Imaging Sciences International Inc, Hatfield, PA, USA) in “Extended Field” modus (FOV: 16 cm diameter/22 cm height; scan time: 2 × 20 s; voxel size: 0.4 mm). Patients were scanned while seated in natural head position. They were asked to swallow, to relax their lips and facial muscles and to keep their eyes open. The acquired CBCT data were exported in DICOM format and imported into Maxilim® software (Medicim NV, Mechelen, Belgium).

### Surgical planning

In Maxilim®, a 3D virtual augmented head model was rendered and positioned in a reference frame as described by Swennen *et al*.^16^ Subsequently, virtual osteotomies were performed to simulate the Le Fort I and BSSO osteotomies.

The maxillary and mandibular segments were positioned to the desired positions to create a harmonious 3D soft tissue facial profile, as simulated in real-time by the Maxilim software using the mass tensor model based soft tissue simulation. If required, an additional virtual chin osteotomy was simulated. Based on the 3D virtual planning, one intermediate and one final interocclusal splint were milled to transfer the virtual planning to the patient in the operating theatre.

Between 2010–2012 the clinical protocol for bimaxillary osteotomy was to start with the BSSO that was followed by the Le Fort I osteotomy (mandible-first). After 2012 this protocol was changed, and the Le Fort I was performed prior to the BSSO (maxilla-first).

### Surgical procedure

All bimaxillary osteotomies were performed or supervised by one experienced surgeon (MdK). After nasotracheal intubation, the mucobuccal fold of the maxilla and the mandibular ramus regions were infiltrated with local anaesthetic (Ultracain Ds-Forte). In cases of mandible-first procedure, a BSSO was performed according to the Hunsuck modification^17^. After the completion of the osteotomies using osteotomes, the distal segment of the mandible was placed in the planned position using the prefabricated interocclusal intermediate splint and stabilized with intermaxillary fixation (IMF). The proximal segments were gently pushed backward and upward to seat the condyles. The mandibular segments were fixed with two titanium miniplates (one on each side) and monocortical screws (Champy 2.0 mm, KLS Martin, Tuttlingen, Germany). Following the BSSO, a Le Fort I procedure was performed. After an incision in the gingivobuccal sulcus and elevation of mucoperiosteum and nasal mucosa, the osteotomies were made with a reciprocal saw at the Le Fort I level. The lateral nasal walls and nasal septum were osteotomized with nasal osteotome. The piriform aperture and nasal spine were rounded. After mobilization of the maxilla, it was positioned in the planned position using a prefabricated final interocclusal splint. Fixation was performed with four 1.5 mm miniplates (KLS Martin, Tuttlingen, Germany) and 4 mm screws, one paranasal and one on the maxillary buttress on each side. Alar cinch suture and VY sutures were used accordingly. The mucosa was closed with a 3–0 Vicryl suture (Ethicon, Johnson and Johnson Medical, Norderstedt, Germany). Depending on the stability of the occlusion, the interocclusal splint was left in place and tight elastics were used in the first week after surgery. Guiding elastics were applied after the first week, in conjunction with the postoperative orthodontic treatment. In cases of maxillary first procedure, the Le Fort I osteotomy was carried out first, after which the BSSO was performed. The surgical protocol and method of fixation were identical as described in the mandible-first procedure.

### 3D analysis of 3D planned and actual postoperative positioning of jaws

The accuracy of the postoperative surgical result was compared to the virtual planning and evaluated using the following steps.

Step 1: The 3D rendered pre- and postoperative 3D virtual head models were aligned by using voxel-based registration upon the anterior cranial base^18, 19^.

Step 2: Virtual triangles were constructed on the maxilla and distal mandibular segment by using previously validated cephalometric landmarks (Table 1)^8, 20^.

**Table 1:** Definitions of the 3D cephalometric landmarks.

Reference landmarks	Description of landmarks	**Bilateral**
Nasion (N)	The midpoint of the frontonasale suture.	
Sella (S)	The center of the hypophyseal fossa.	
Porion (Por)	The most superior point of the meatus acusticus e-ternus.	X
Orbitale (Or)	The most inferior point of the orbital rim.	X
**Landmarks maxilla**		
Upper incisor (UI)	The most mesial point of the incisor edge of the right upper central incisor.	
Mesial cusp 16	The most inferior point of mesial cusp of the crown of the right first upper molar.	
Mesial cusp 26	The most inferior point of mesial cusp of the crown of the left first upper molar.	
**Landmarks mandible**		
Lower incisor (LI)	The most mesial point of the incisor edge of the left lower central incisor.	
Mesial cusp 36	The most superior point of mesial cusp of the crown of the left first lower molar.	
Mesial cusp 46	The most superior point of mesial cusp of the crown of the right first lower molar.	

Step 3: The preoperative virtually osteotomized maxilla and distal mandibular segment were translated to the 3D planned position in Maxilim® by voxel-based registration. The landmarks placed on the preoperative maxilla and mandible, and thus the previously constructed triangles were translated along with the maxilla and mandible to the 3D planned position. The coordinates of the triangles were imported into the OGA to compute the 3D planned sagittal, vertical and transverse translations as well as rotations (pitch, roll and yaw) of the maxilla and distal mandibular segment.

Step 4: The maxilla and mandibular segments were again translated from the 3D planned position to the postoperative position through voxel-based registration that resulted in a displacement of the virtual triangle. The coordinates of the landmarks (virtual triangle) in the postoperative position were imported into the OGA. The translation and rotation differences of the maxilla and distal mandibular segment between the 3D planning and actual postoperative results were calculated^8^.

### Statistical analysis

Statistical data analyses were performed with SPSS 22.0.1 (IBM Corp., Armonk, NY, USA). The mean and absolute mean differences between the 3D planning and postoperative results were calculated. Analysis of variance (ANOVA) was used to test for differences in postoperative results and the planning between the maxillary first and mandible-first groups with correction for possible confounding factors at the 5% level of significance (p ≤ 0.05). Univariate and multivariate regression analysis were applied to identify the prognostic factors that influence the postoperative result.

### Data availability statement

The datasets generated during and/or analysed during the current study are available from the corresponding author on reasonable request.
